# Toward a more accurate 3D atlas of *C. elegans* neurons

**DOI:** 10.1186/s12859-022-04738-3

**Published:** 2022-05-28

**Authors:** Michael Skuhersky, Tailin Wu, Eviatar Yemini, Amin Nejatbakhsh, Edward Boyden, Max Tegmark

**Affiliations:** 1 Department of Brain and Cognitive Sciences, Massachusetts Institute of Technology, Cambridge, USA; 2 Department of Computer Science, Stanford University, Stanford, USA; 3 Department of Neurobiology, University of Massachusetts Chan Medical School, Worcester, USA; 4 Department of Neuroscience, Columbia University, New York, USA; 5 Zuckerman Institute, Center for Theoretical Neuroscience, Columbia University, New York, USA; 6 Howard Hughes Medical Institute, Chevy Chase, USA; 7 Department of Physics, Institute for Brains, Minds and Machines, Institute for Artificial Intelligence and Fundamental Interactions, Massachusetts Institute of Technology, Cambridge, USA

**Keywords:** *Caenorhabditis elegans*, Neuron identification, Point-cloud alignment, Cell atlas

## Abstract

**Background:**

Determining cell identity in volumetric images of tagged neuronal nuclei is an ongoing challenge in contemporary neuroscience. Frequently, cell identity is determined by aligning and matching tags to an “atlas” of labeled neuronal positions and other identifying characteristics. Previous analyses of such *C. elegans* datasets have been hampered by the limited accuracy of such atlases, especially for neurons present in the ventral nerve cord, and also by time-consuming manual elements of the alignment process.

**Results:**

We present a novel automated alignment method for sparse and incomplete point clouds of the sort resulting from typical *C. elegans* fluorescence microscopy datasets. This method involves a tunable learning parameter and a kernel that enforces biologically realistic deformation. We also present a pipeline for creating alignment atlases from datasets of the recently developed NeuroPAL transgene. In combination, these advances allow us to label neurons in volumetric images with confidence much higher than previous methods.

**Conclusions:**

We release, to the best of our knowledge, the most complete full-body *C. elegans* 3D positional neuron atlas, incorporating positional variability derived from at least 7 animals per neuron, for the purposes of cell-type identity prediction for myriad applications (e.g., imaging neuronal activity, gene expression, and cell-fate).

**Supplementary Information:**

The online version contains supplementary material available at 10.1186/s12859-022-04738-3.

## Background

The nematode Caenorhabditis elegans (*C. elegans*) is among the most-studied animals in neuroscience, and remains the only multicellular organism with a fully mapped connectome. Capable of exhibiting complex behaviors despite having only several hundred neurons, it has provided an abundance of neuroscientific insights. The goal of this paper is to further improve the scientific utility of *C. elegans* by enabling more accurate and automated identification of its neurons, that are either unlabeled, or have been labeled by one or more colors that encode information regarding identity.

### Limitations of the original *C. elegans* connectome

The electron micrograph (EM) reconstruction of the *C. elegans* nervous system and its connectome were first fully described in a seminal 1986 paper [1]. This was an invaluable technical achievement requiring a decade of work to hand-trace every neuron and connection from the EM sections. Due to technical limitations involved with preparing worm samples, this nervous system reconstruction was derived from a mosaic of overlapping sections from five individual worms. These five worms consist of three adult hermaphrodites, a fourth larval stage (L4) animal, and one adult male. Thus, in combination, this reconstruction provides a generalized view of the worm’s nervous system.

While a generalized view of the worm’s nervous system has proven valuable to the field, it lacks representation for the idiosyncrasies found among individual worms. Moreover, preparations for EM imaging can introduce non-linear distortions. Preparing worms for EM imaging requires that they be first physically sliced open so that the fixative may bypass the impermeable cuticle [2, 3]. As the worm interior is under a higher pressure, breaching the cuticle results in morphological changes, and the commonly used EM fixative osmium tetroxide has also been shown to alter morphology [4]. In the original reconstruction of the worm nervous system, animals were serially sectioned into approximately 50 nm thick slices. The combination of these steps yielded distortions that required correction when unifying worm sections into a general visual representation of its nervous system. As a result, substantial manual correction was introduced when generating this canonical nervous system illustration. These corrections and the multi-animal synthesis thus present a generalized view of the worm’s nervous system, albeit one that lacks quantification of neuron positions and measurements of their variability to recapitulate individual idiosyncrasies present within the population.

### Existing *C. elegans* atlases

**Fig. 1:**
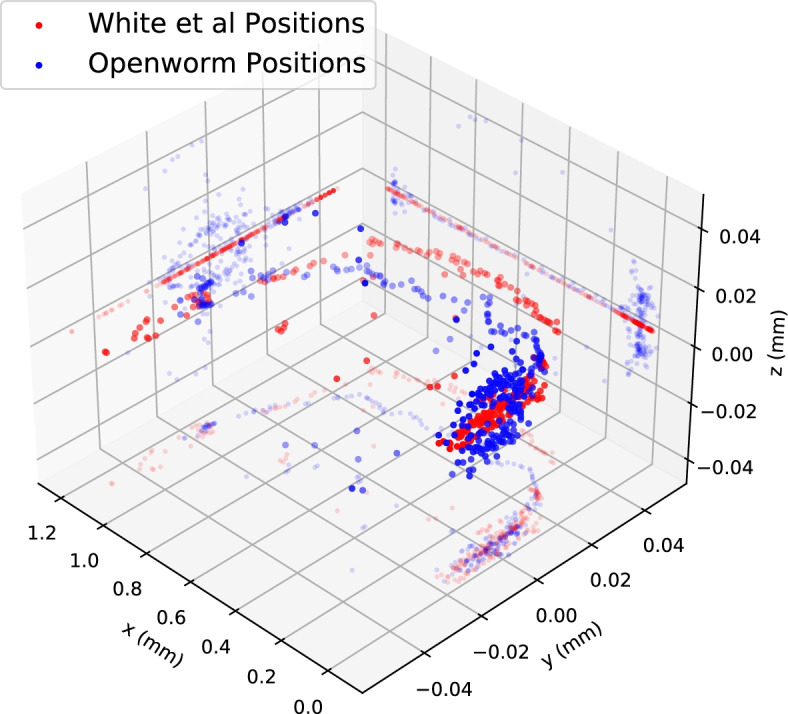
*C. elegans* atlases from White et al. (red) and OpenWorm (blue), after straightening and uniform scaling using a corrected version of [21], with axial projections, unequal scaling

This illustrative 1986 worm atlas has often been treated as canon, or at least as an atlas of sufficient quality to compare with contemporary data from modern and more reproducible measurement techniques. This is in large part due to it being the only atlas of its kind up until very recently [5, 6]. For example, Scholz et al. [7] aligns this atlas to fluorescent imaging data to assign neuron identity. Unfortunately, using this generalized illustrative atlas for neural identification purposes can lead to unlabeled and even mislabeled neurons. This is largely due to the density of neurons in various ganglia and ambiguities in atlas matching that are present as a result. In addition to these limitations and those listed in the previous section, a 2004 version of this atlas, commonly used in papers that make use of *C. elegans* neuron positions, was produced by further processing the original version so as to translate the 1986 illustration into semi-quantifiable measurements. This version was produced by Choe et al. [8, 9] and popularized by Kaiser et al. [10]. It tabulates neural positions that were measured by directly scanning and tracing the physical 1986 paper. The 2004 atlas (Fig. 1) consists of only 277 of the 302 neurons found in adult hermaphrodites. Crucially, due to the 2D geometry of the scanned and processed original paper, the 2004 atlas is also 2D. The third dimension in modern 3D datasets is therefore typically discarded before matching against this 2D atlas and may further increase the misidentification rate. In total, these limitations motivate the creation of a representative 3D atlas of *C. elegans* neuron positions and their variability.

In the decades since the original EM connectome was published [1], many individual *C. elegans* neurons have been further studied and characterized. Their updated information has been incorporated into the widely used *C. elegans Atlas* [11], and the contents of this reference text have been artistically assembled into the popular and influential OpenWorm project [12, 13]. OpenWorm presents information for individual neurons and their connectivity, assembled into a 3D approximation of an adult hermaphrodite worm. However, the inherent positional variability of neurons in the head and tail is now readily apparent from multicolor *C. elegans* strains, designed for neural identification, that were used to measure neuron positions and their variability across multiple animals [5, 6, 14]. These strains and concurrent algorithmic advances demonstrate how fluorescent-protein barcodes can be used to accurately determine neuron identities in volumetric images [15–20].

The strong interest in *C. elegans* research coupled with great recent progress in all aspects of *C. elegans* imaging makes it timely to develop improved tools for whole-nervous-system *C. elegans* neuron identification and make them available to the community. This is the goal of the present paper. The rest of this paper is organized as follows: we present our improved alignment method in “Methods” together with a pipeline for creating improved atlases of whole-worm neuron positions. We then compare the neuron identification accuracies of various methods in “Results”, applying them to simulated data and then to our new atlas. We then summarize these results in our “Conclusion”.

## Methods

### New NeuroPAL-derived atlas

**Fig. 2:**
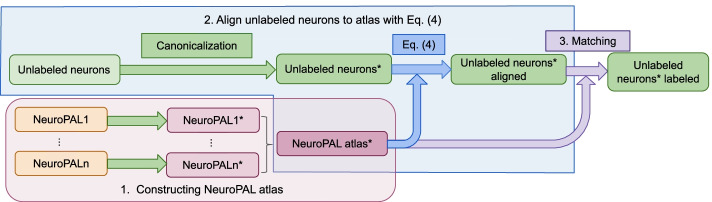
Diagram of our NeuroPAL atlas construction and alignment pipeline, with each algorithm labeled. The * denotes the neuron positions after canonicalization

Our construction of the NeuroPAL atlas consists of three steps:

1. Preparing and imaging NeuroPAL worms and turning each image into a pair of measured quantities as in “Preparing and imaging the NeuroPAL worms”: a point cloud (a position vector and optional color information for each neuron) and a hull outline (a smooth closed 2D curve).
2. Canonicalizing each pair by normalizing the hull into a standard straightened hull, along with the point cloud in the associated canonical 3D space.
3. Combining point clouds from multiple worms into a single point cloud atlas (Fig. 2).

#### Preparing and imaging the NeuroPAL worms

Brainbow [22] is a stochastic technique that has been used to differentiate individual neurons from neighboring ones by expressing unique ratios of red, green, and blue fluorescent proteins. Unfortunately, Brainbow coloring is generated randomly and thus the colors cannot be used to identify neuron types. In contrast, the recent NeuroPAL *C. elegans* transgene introduces an alternative deterministic technique that reveals the unique identity of each individual neuron, at all larval stages of both worm sexes (hermaphrodites and males) [6, 23]. NeuroPAL worms have an identical and invariant colormap by way of the stereotyped expression of four distinguishable fluorophores. These four fluorophores leave the green channel free, and thus it can be used to map gene expression using the common GFP, CFP, or YFP based gene reporters, or to measure dynamic neural activity using the GCaMP reporter.

A complicating factor in position-derived identification is that individual neuron positions are known to be locally variable between different worms [5, 6, 11]. This is caused partly by *C. elegans* size and shape differences, and partly because of inherent positional variance that may be so extreme as to have nearby neurons switch relative positions.

Adult *C. elegans* were imaged in accordance with the methods as described in [6], and the images were processed with a semiautomatic method using accompanying software of [6]. Neuron positions were detected by the software, and any required corrections were made manually. The software predicted neuron identities for the head and tail of each worm, and these were manually checked and relabeled as needed. Since there were no prior statistical atlases for neurons in the worm midbody, the identities of these neurons were manually annotated for each worm. The result was a labeled point cloud for each individual corresponding to the neuron positions.

The original 1986 nervous system reconstruction [1] was assembled using a mosaic of 5 overlapping image sections, each corresponding to part of the worm, that when pieced together would represent entire nervous system. Each section was taken from one of 5 worms representing a mixture of age and sex: 3 adult hermaphrodites, 1 L4, and 1 adult male. In our atlas, to maintain a generalized representation of the nervous system we used 7 worms: 1 adult hermaphrodite, 4 young-adult hermaphrodites, 1 L4 hermaphrodite, and 1 adult male. Here, each individual worm has neuron positional information represented for its whole body, rather than only a body section, thus contributing a holistic representation of the nervous system from each of these animals. All 7 worms were positioned on an agar pad for imaging such that their left-right axis extended between the glass coverslip and slide. As a result, the worm samples and representative left-right axis may have been slightly compressed between these two surfaces. Similarly, the dorsal-ventral and potentially the anterior-posterior axes may have been slightly elongated as a result of this compression.

#### Straightening method

For successful alignment, canonicalization is essential in ensuring that the neuron point clouds from different worms, that were imaged in different postures, orientations, and morphology, lie in the same canonical space to provide a reasonable starting point for neuron matching. Mathematically, canonicalization corresponds to a continuous and invertible 3D mapping that that gives all imaged worm hulls the same shape regardless of the proportions and bending state of the worms.

Several methods have been previously used for this problem of straightening worm hulls for eventual canonicalization [24, 25]. Because worms are imaged under a coverslip and thus lie in a 2D plane, such methods customarily model the 3D mapping as a 2D mapping, leaving the vertical dimension unchanged. We make the same simplification, because our current data does not provide sufficiently accurate 3D hull determination (worm edges are too blurred in less-focused horizontal slices far above or below the midplane).

Most previous methods do not attempt to preserve volume, and pose challenges related to distorted straightening at the head and tail. This is unfortunate, since these dense areas, presumably responsible for a majority of the worm’s information processing, are the most important to get right for scientifically-relevant neuron identification. For example, we originally tested a simple 2D canonicalization where the new *x*-coordinate was defined as the distance along the worm midline estimated by skeletonizing the hull image, and the new y-coordinate was defined as the perpendicular distance to this midline. This scheme unfortunately resulted in problematic volume distortions associated with the contorted morphology of an unrestrained worm, and produced relatively poor neuron matching accuracy.

**Fig. 3:**
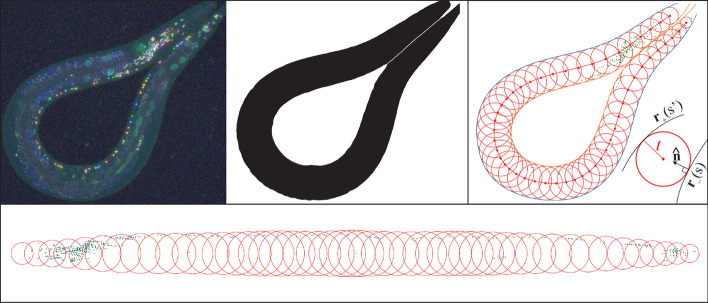
Starting with a worm image (top left), we first determine the hull (top center). Our algorithm then inscribes 1,000 tangent circles (top right) in the hull (for clarity, only 50 circles are shown here). Finally, the circle sequence is straightened, which defines the canonicalization mapping of their inscribed neuron positions (bottom)

We therefore propose a worm canonicalization method which produces more consistent and biologically realistic point clouds. As explained below, the 2D mapping can be geometrically interpreted by filling the worm with inscribed circles as illustrated in Fig. 3. We first make a hull map for each worm, by binarizing the slice from the *C. elegans* *z*-stack that represents the largest-area 2D hull of the worm (Fig. 3, middle panel).

We then split the worm hull boundary into two parameterized curves representing opposite edges, ${\mathbf{r}}_-(s)$ and ${\mathbf{r}}_+(s')$, where $s\in [0,1]$ and ${\mathbf{r}}_-(s)$ runs clockwise from head to tail as *s* increases. We define these curves by cubic spline fits to the binarized hull image. As illustrated in Fig. 3 (right panel), the distance from the point ${\mathbf{r}}_+(s')$ on one edge of the worm to a circle of radius *t* tangent to ${\mathbf{r}}_-(s)$ on the other edge is then given by

1 $$\begin{aligned} d(s,s',t)\equiv \left| {\mathbf{r}}_-(s) + \hat{\varvec{n}}_-(s)t - {\mathbf{r}}_+(s') \right| - t, \end{aligned}$$

where $\hat{\varvec{n}}_-(s)\equiv (-\dot{r}_y(s),\dot{r}_x(s))/|{\dot{\mathbf{r }}}(s)|$ is the unit inward tangent vector at ${\mathbf{r}}_-(s)$, the vector ${\mathbf{r}}_-(s) + \hat{\varvec{n}}_-(s)t$ is the center of the aforementioned circle, and dots denote derivatives with respect to *s*. By numerically minimizing over $s'$, we find the distance *d*(*s*, *t*) from the circle to the opposite worm edge:

2 $$\begin{aligned} d(s,t)\equiv \min _{s'} d(s,s',t). \end{aligned}$$

We numerically solve the equation $d(s,t)=0$ to determine the radius *t*(*s*) where the circle is tangent to both worm edges. The variable $s\in [0,1]$ thus parametrizes a continuous family of circles of radius *t*(*s*) centered at

3 $$\begin{aligned} {\mathbf{r}}_0(s)\equiv {\mathbf{r}}_-(s) + \hat{\varvec{n}}_-(s)t(s). \end{aligned}$$

Finally, these circles are used to remap the *C. elegans* neurons using the methods illustrated in Fig. 3. For each worm, 1000 equally spaced circles are inscribed. All worm-inscribed circles are then translated so that their midpoints lie on the *x*-axis, retaining the distances between adjacent circle midpoints, and rotated so that the worm midline corresponds to the x-axis (Fig. 3, bottom). Each circle thus defines an affine transformation into the canonical space. Each neuron is mapped into this canonical space by applying the affine transformation for each of the circles that inscribe it, and averaging the result. Finally, the straightened group of neurons are isotropically rescaled so as to occupy $x\in [0\,\upmu{\text{m}}, 800\,\upmu{\text{m}}]$.

**Fig. 4:**
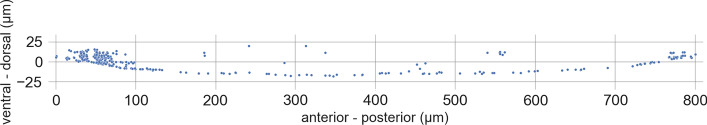
Sagittal view of our NeuroPal-derived atlas

**Fig. 5:**
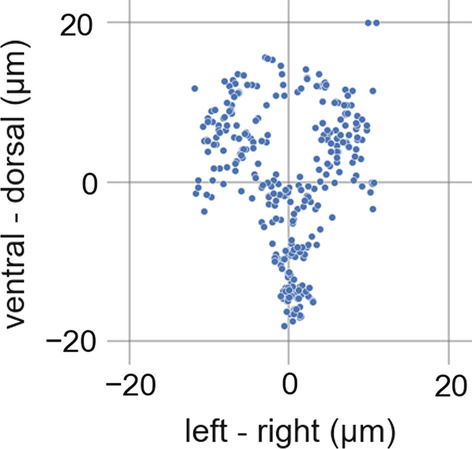
Transverse view of our NeuroPal-derived atlas

#### Atlas construction

After applying our canonicalization procedure to each individual worm and obtaining corresponding 3D neuron point clouds for each one, we combine these point clouds into a single NeuroPAL-derived whole worm atlas by using the median position coordinates for each neuron. We use the median rather than the mean since it is more robust to outliers and resistant to overall shrinking. To further improve the atlas quality in the neuron-dense head and tail regions, which are relatively rigid compared to highly deformable midbody, we rigidly align the high resolution head and tail atlases from [6](which were based on 10 worms) to the two point clouds consisting of their corresponding neurons taken from our straightened 7-worm whole-body atlas, and finally combine these neuron positions with the original midbody positions to obtain our final atlas.

This resultant atlas is illustrated in Figs. 4 and 5, and the 300 canonicalized neuron position coordinates are listed in Additional file 1: S2 Appendix. We do not provide data for the two CAN cells in our dataset, as previous broad investigations of panneuronal markers found none that solely express in neurons and also express in CAN [26]. All panneuronal markers that expressed in CAN also expressed in non-neuronal tissues such as epithelium, intestine, glands, or muscle. Non-neuronal tissues are often larger than neurons and fluorescent expression within them can can occlude neuronal imaging. Thus, to date, all whole-brain activity imaging as well as neural identification strains have used panneuronal markers that exclude CAN and, accordingly, these two cells are not represented in our dataset.

### New alignment technique

We now turn to the challenge of aligning the neuron point cloud from an observed worm to a known atlas, so as to determine the likely identity of each observed neuron.

#### Existing coherent point drift alignment

Coherent Point Drift [27] (CPD) is a probabilistic alignment method for sparse point clouds that has been extensively used to align *C. elegans* neuron positions [5, 7, 19]. CPD represents the first point set by Gaussian mixture model (GMM) centroids and aligns to a second point set by maximizing the likelihood while forcing the points to move coherently as a group. In the non-rigid case, this is implemented as a motion over time guided by a velocity field determined by maximizing the GMM likelihood penalized by motion incoherence. CPD performs well on our *C. elegans* problem when the two point clouds undergo rigid-body rotation, translation and scaling, but as we will show below, has limited robustness in cases of cropping, size imbalance, and realistic biological deformation.

#### Colors

Since NeuroPAL is a fluorescent-labeling transgene that improves neuron distinguishability using several colors, it is highly desirable to exploit this color information to improve neuron matching. This begs the question: how many colors are needed for accurate alignment? With better alignment methods, are fewer colors sufficient? To quantify the relationship between color and accuracy, we report below a series of simulation results where each neuron is randomly assigned a simulated color.

#### Novel generalized-mean alignment

To improve the robustness of the alignment and address the issues in CPD, we introduce a novel *Generalized-Mean* (GM) alignment algorithm. For labeled neuron positions $\mathbf {r}^{(1)} = \{ \mathbf {r}_i^{(1)} \}_{i=1}^{n_1}$ from reference worm 1 and unlabeled neuron positions $\mathbf {r}^{(2)} = \{ \mathbf {r}_j^{(2)} \}_{j=1}^{n_2}$ from worm 2 ($n_k$ denotes the number of neurons in the $k^\mathrm{th}$ worm), and optionally provided indexed colors $\mathbf {c}^{(1)} = \{c_i^{(1)}\}_{i=1}^{n_{1}}$ and $\mathbf {c}^{(2)} = \{c_j^{(2)}\}_{j=1}^{n_{2}}$, we introduce the following loss function to minimize by way of gradient descent on the $\mathbf {r}^{(2)}$ positions:

4 $$\begin{aligned} &\ell_{GM}(\mathbf {r}^{(1)}, \mathbf {r}^{(2)},\mathbf {c}^{(1)},\mathbf {c}^{(2)})= \sum_{j=1}^{n_2}\left( \frac{\sum_{i=1}^{n_1} \delta_{c_i^{(1)} c_j^{(2)}} \left| \mathbf {r}^{(1)}_i-\mathbf {r}^{(2)}_j\right| ^\gamma }{\sum_{i=1}^{n_1} \delta_{c_i^{(1)} c_j^{(2)}} }\right) ^{\frac{1}{\gamma }} \end{aligned}$$

where $|\mathbf {r}_i^{(1)}-\mathbf {r}_j^{(2)}|$ is the Euclidean distance between $\mathbf {r}_i^{(1)}$ and $\mathbf {r}_j^{(2)}$, and the Kronecker delta $\delta_{c_i^{(1)} c_j^{(2)}}=1$ if the two colors are equal, vanishing otherwise. The generalized mean is defined by the hyperparameter $\gamma <0$ which, as explained in [28], encourages pairing. More specifically, for each unlabeled neuron positions $\mathbf {r}_j^{(2)}$, we have a generalized-mean of its distance to all the labeled neuron positions $\mathbf {r}_i^{(1)}$ that have the same color (as enforced by $\delta_{c_i^{(1)} c_j^{(2)}}$). When $\gamma <0$, the smaller the distance, the larger $\left| \mathbf {r}^{(1)}_i-\mathbf {r}^{(2)}_j\right| ^\gamma$ is. Furthermore, as proved in [28], this generalized-mean loss has the property that the smaller $\left| \mathbf {r}^{(1)}_i-\mathbf {r}^{(2)}_j\right|$ is among all the $\mathbf {r}^{(1)}$, the larger the gradient is to drive them closer. Here $\gamma$ tunes how much the algorithm is focusing on the smaller distances. For example, when $\gamma \rightarrow -\infty$, Eq. (4) reduces to $\sum_{j=1}^{n_2}\min_{\delta_{c_i^{(1)}c_j^{(2)}=1}} \left| \mathbf {r}_i^{(1)}-\mathbf {r}_j^{(2)}\right|$ where each unlabeled $\mathbf {r}_j^{(2)}$ only focuses on the nearest labeled $\mathbf {r}_i^{(1)}$ which can easily fall into a local minimum. However, allowing a *finite* negative $\gamma$ also allows consideration of other potential parings that are not as near. In this paper, we set $\gamma =-6$, which we experimentally found to achieve a good balance between the aforementioned pairing effect and the desire for error correction whereby neurons at slightly larger distance produce enough of a gradient to be pushed together, which is crucial for avoiding getting trapped in suboptimal local minimal during the initial stages of training. In contrast, $\gamma =2$ would correspond to $\ell$ being minus two times the log-likelihood of a Gaussian mixture model, making the loss similar to that of CPD with a Gaussian kernel.

### Algorithm testing framework

To identify unknown neurons, our pipeline proceeds as illustrated in Fig. 2. It first canonicalizes the unlabeled neurons, then aligns them with the canonicalized atlas as described above. The alignment function takes as input an unlabeled point cloud of neuron positions, an atlas point cloud with known neuron IDs (with optional neuron colors), and returns transformed positions for the unlabeled point cloud (step 2). Finally, in step 3, each neuron with transformed position is assigned an ID by the following procedure: for the $n_2\times n_1$ matrix of pairwise Euclidean distances between the $n_2$ neurons in the unlabeled point cloud and the $n_1$ neurons in the atlas, find the smallest element in the matrix, assign the corresponding ID, then delete this row and column in the matrix, and repeat. If colors are provided, each unlabeled neuron can only be assigned to a neuron in the atlas with the same color, so we choose the smallest element with unlabeled colors at each iteration.

We quantify the accuracy of all algorithms by testing them on simulated data where the ground truth is known. For these simulations, we use the OpenWorm dataset as the ground truth point cloud, distort it with simulated noise and biological deformations as described below, and finally measure which algorithms provide the most accurately reconstructed neuron identifications.

### Parameterizing more realistic worm deformation

Working in the above-defined canonical space, we express the relation between the neuron positions ${\mathbf{r}}_i^{(1)}$ in reference worm 1 (our atlas, say) and observed neuron positions ${\mathbf{r}}_i^{(2)}$ for a worm 2 as

5 $$\begin{aligned} {\mathbf{r}}_i^{(2)} = {\mathbf{r}}_i^{(1)} + {\mathbf{f}} \left( {\mathbf{r}}_i^{(1)}\right) , \end{aligned}$$

for a *deformation function* ${\mathbf{f}} (r)$ that may include a random component. A very easy-to-simulate type of deformation is to simply add independent Gaussian noise to all coordinates of all neurons. This corresponds to treating all neuron positions ${\mathbf{r}}_j^{(2)}$ as independent parameters, and is tantamount to ignoring all biological constraints on tissue stretching.

We wish to regularize the problem to limit our analysis to more biologically plausible deformations, reflecting the known fact that positional variation between organisms exhibits correlation, whereby the deformation vector ${\mathbf{f}}$ is typically similar in direction and magnitude for adjacent neurons. In other words, we wish the deformation function ${\mathbf{f}} ({\mathbf{r}})$ to be relatively smooth, corresponding to nearby parts of the worm mainly shifting together as a coherent unit. We model the deformation field as a Gaussian mixture:

6 $$\begin{aligned} {\mathbf{f}} ({\mathbf{r}})=\sum_{n=1}^N \mathbf{d}_n e^{-\frac{|{\mathbf{r}}-{\mathbf{r}}_n|^2}{2\sigma ^2}} \end{aligned}$$

**Fig. 6:**
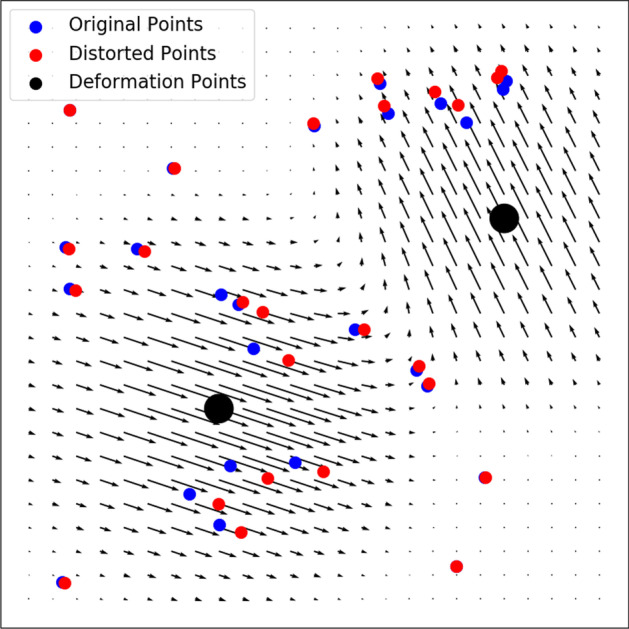
Illustration of simulated biological noise simulated with the “bio-realistic” deformation field

Here the deformation function is parametrized by a vector $\mathbf{p}$ of $6N+1$ parameters: the number $\sigma$, the components of the displacement vectors $\mathbf{d}_n$, and the displacement centers ${\mathbf{r}}_n$. A sample deformation field is visualized in Fig. 6. For better numerical stability, we add three redundant parameters in the form of an overall global displacement vector $\mathbf{s}$ added to ${\mathbf{r}}_n$. This deformation field is added as a term in our GM loss function, to create our “GM Realistic” algorithm.

### Hyperparameter tuning

The performance of all tested alignment algorithms is highly hyperparameter dependent. To make a robust comparison of methods, we must ensure that for each algorithm we are using the optimal parameters for our use case. Therefore, we submit each algorithm to parallelized hyperparameter optimization.

Using the “Random Search” followed by the “Local Search” algorithms in the Sherpa [29] parameter tuning package, input parameters were tuned until alignment accuracy did not improve further. Here, alignment accuracy was defined as the average of performance per method in the tests “Robustness to Cropping”, “Robustness to Dropout”, and “Robustness to Biological Noise”. The determined optimal parameters (Additional file 1: S1 Appendix) for each algorithm were used in all subsequent testing.

## Results

We now compare the performance of our atlas and alignment methods with existing techniques. We designed experiments to answer the following questions: Firstly, how does our alignment method compare with existing methods against various types of adverse point cloud distortions? This is answered in Alignment performance on simulated data. Secondly, how does the new atlas perform compared to existing atlases? We answer this question in Testing alignment methods - Real Data by comparing the accuracy of aligning unlabeled neurons to existing and new atlases. Thirdly, since NeuroPAL and other labelling schemes use multiple colors to reveal identification, how many colors are necessary to achieve acceptable accuracy? In Testing alignment methods - Real Data, we also examine the effects of multiple colors on identification accuracy.

The following tests were devised: the point cloud positions of the 302 Openworm neurons were taken as a test set, as it can be assumed that these positions are reasonably representative of the morphology of actual *C. elegans* neurons [13]. Then, a copy of this point cloud was made, and selected perturbations, designed to simulate real world experimental conditions were performed. Finally, the group of points was randomly perturbed by a single vector drawn from a uniform distribution between $\pm 5$ microns in each of the *x*, *y*, and *z* directions. The alignment algorithms were then used to align the perturbed neuron point cloud to the original one. We test alignment with GM Realistic (GM with Gaussian deformation centers as defined in Eq. (6), to account for group neuron movement), CPD Rigid (CPD with rigid transformation, as defined in Fig. 2 of [27]), and CPD Deformable (CPD with deformable transformation, as defined in Fig. 4 of [27]).

This procedure was repeated 40 times at each perturbation setting, with resulting accuracies averaged together to provide a final metric of accuracy.

### Alignment performance on simulated data

To simulate point clouds data similar in structure to those from real *C. elegans* images, we start with the OpenWorm atlas [13] and generate a point cloud of neuron positions with the ground-truth neuron IDs hidden from the alignment methods. A good alignment and identification pipeline should be robust to the above scenarios; therefore we sought to test the robustness of our alignment algorithm when deforming the Openworm point cloud dataset in these ways. In the following plots, results for optimally tuned parameters are shown.

#### Robustness to cropping

Commonly, *C. elegans* activity research focuses on the head of the worm, due to microscope field of view or resolution reasons, or hypotheses aimed at the nerve ring. During alignment, the head neurons are usually matched to a larger atlas. Therefore, a good identification method must perform well when aligning a dataset of unlabeled neurons that may reference a cropped subset of the comparison atlas.

For the first test, we start with a full Openworm point cloud that has been isotropically rescaled so as to occupy $x\in [-600\,\upmu {\text{m}}, 200\,\upmu {\text{m}}]$. We choose all head neurons (located at $x>0\,\upmu {\text{m}}$) as the unlabeled neuron set, and also create copies of the Openworm point cloud with various *x* cropping thresholds to act as the comparison atlas. We perturb the larger atlas as a rigid group away from the head neurons by a single vector drawn from a uniform distribution between $\pm 5$ microns in each of the *x*, *y*, and *z* directions. Then we use the alignment algorithms to align the unlabeled head neurons to the various croppings of the larger atlas, to test how different algorithms are robust to the imbalance of the two neuron sets.

**Fig. 7:**
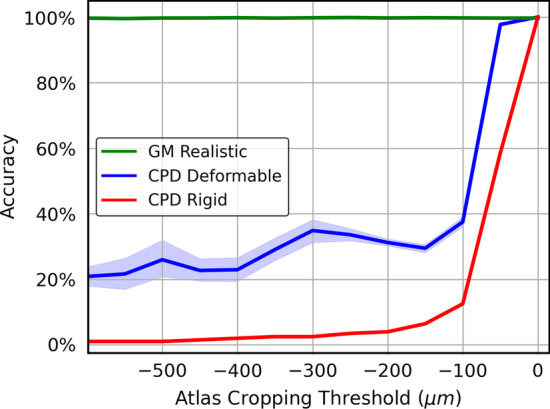
Cropping Performance (40 run average): Alignment accuracy after aligning OpenWorm data cropped to the head to OpenWorm data cropped at various *x*-coordinates and perturbed as a group in a random direction in *x*, *y*, and *z* each at up to 5 microns. At $x=0$, the atlas cropping isolates the head

Fig. 7 shows the resulting alignment accuracy for GM Realistic, CPD Rigid, and CPD Deformable. We can see that the GM Realistic algorithm performs perfectly across all levels of imbalance, while the CPD methods’ accuracy drops quickly when the atlas extends beyond $-100\,\upmu {\text{m}}$. The robustness of our GM method comes from the fact that the loss in Eq. (4) has the soft “clamping” effect that is able to focus on well-matched pairs of neurons while ignoring pairs that are matched poorly. The CPD methods, in contrast, are more sensitive to those badly-matched pairs, so the performance quickly drops when the two neuron sets are imbalanced. We also became aware of differences in alignment performance when aligning two sets of head neurons, from aligning two sets of whole-body neurons. The whole-body neurons take up space that is elongated along one axis, whereas head neurons occupy a relatively tight spherical volume. Because of this, head-head alignments were found to be more likely to fail by getting stuck in false local minima with the CPD methods.

#### Robustness to dropout

Fluorescent-protein expression that is used to identify neurons can at times be too dim to resolve. As a result of their dimness, these neurons cannot be detected or definitively identified. Such situations can occur due to many reasons, for example fast volumetric imaging methods that necessitate a tradeoff between speed and imaging quality. More generally, dimness is often found due to optical anisotropy present in various volumetric imaging techniques. A good identification method must therefore be robust to various levels of neural dropout.

For this, we start with a full OpenWorm point cloud. Then, we randomly remove a set number of neurons, and set the result as our unlabeled point cloud, with another copy of the OpenWorm point cloud as the comparison atlas. We perturb the larger atlas as a rigid group away from the head neurons by a single vector drawn from a uniform distibution between $\pm 5$ microns in each of the *x*, *y*, and *z* directions. Then we use the alignment algorithms to align the unlabeled set to the complete atlas.

**Fig. 8:**
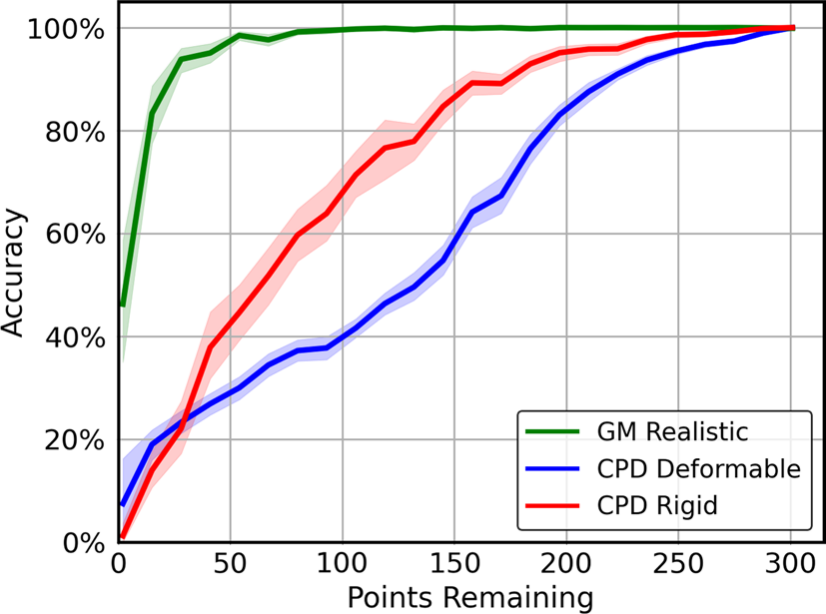
Dropout Performance (40 run average): Alignment accuracy after aligning the OpenWorm atlas to the OpenWorm atlas with random points removed and perturbed as a group in a random direction in x, y, and z each at up to 5 microns

Fig. 8 shows the resulting alignment accuracy for GM Realistic, CPD Rigid, and CPD Deformable. We can see that the GM Realistic algorithm performs the best in all cases of neuron removal, plateauing to 100% accuracy with only 80 out of 300 neurons remaining. The CPD methods are more prone to misidentification by getting stuck in suboptimal local minima.

#### Robustness to biological noise

Imaged *C. elegans* individuals do not typically exhibit identical morphology. For example, an individual that has consumed more food may be larger than another individual of the same age. These morphological differences are not isotropic, but their effects on distortion of adjacent neurons present as collective positional shift, so a good identification method must be robust to such local translation. We simulate such “biological noise” as follows.

We randomly generate *N* deformation points ${\mathbf{r}}_n$ within the volume of the neuron point cloud, and a corresponding displacement vector ${\mathbf{d}}_n$ drawn from a 3D Gaussian distribution of standard deviation $\sigma$, characterizing the “influence radius” of a deformation center, as illustrated in Fig. 6. In other words, each neuron is perturbed according to equation (6), with the only difference that now each ${\mathbf{p}}_n$ and ${\mathbf{r}}_n$ are randomly generated instead of learnable parameters.

This biological noise was applied to our test point cloud according to our realistic noise operation Parameterizing more realistic worm deformation, with $N=100$ deformation centers, amplitude $\left| \mathbf {d}_n \right| = 6.1$ and varying influence radius ${\varvec{\sigma} }$. We then perturb the larger atlas as a rigid group away from the head neurons by a single vector drawn from a uniform distribution between $\pm 5$ microns in each of the *x*, *y*, and *z* directions. Then we use the alignment algorithms to align the unlabeled set to the complete atlas.

**Fig. 9:**
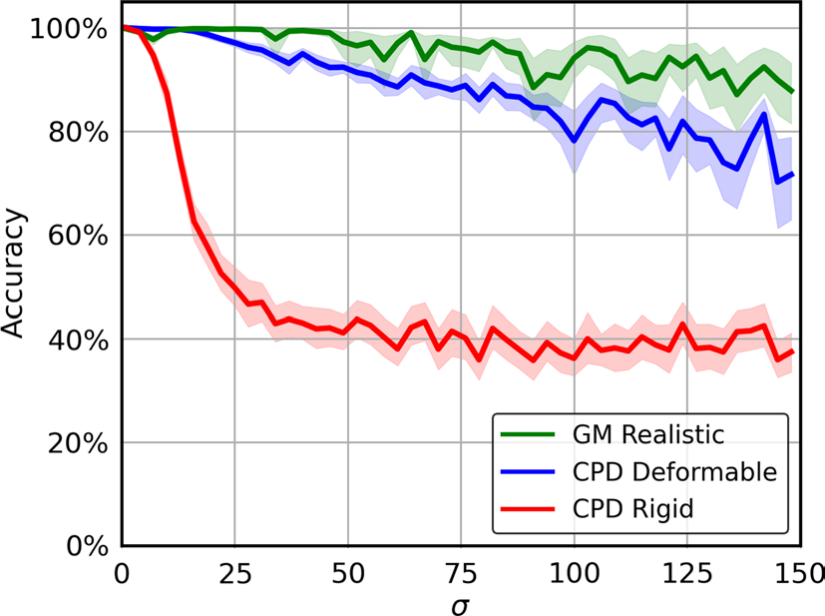
Biological Noise Performance (40 run average): Alignment accuracy after aligning OpenWorm data to OpenWorm data that has been deformed with our biological noise method at various influence radius $\sigma$ and perturbed as a group in a random direction in x, y, and z each at up to 5 microns

In Fig. 9, we can see that the accuracy of CPD Rigid drops rapidly for biological noise influence radius beyond $15\,\upmu{\text{m}}$. This may be because CPD Rigid assumes that all the neurons move as a rigid body that only allows rotation and translation as a whole, which is insufficient to model the realistic biological noise with relative expansion and contraction between neurons. On the other hand, while CPD Deformable performs much better than CPD Rigid, it underperforms GM Realistic across all the tested influence radii of the biological noise.

### Testing alignment methods—real data

#### Atlas performance tests

**Fig. 10:**
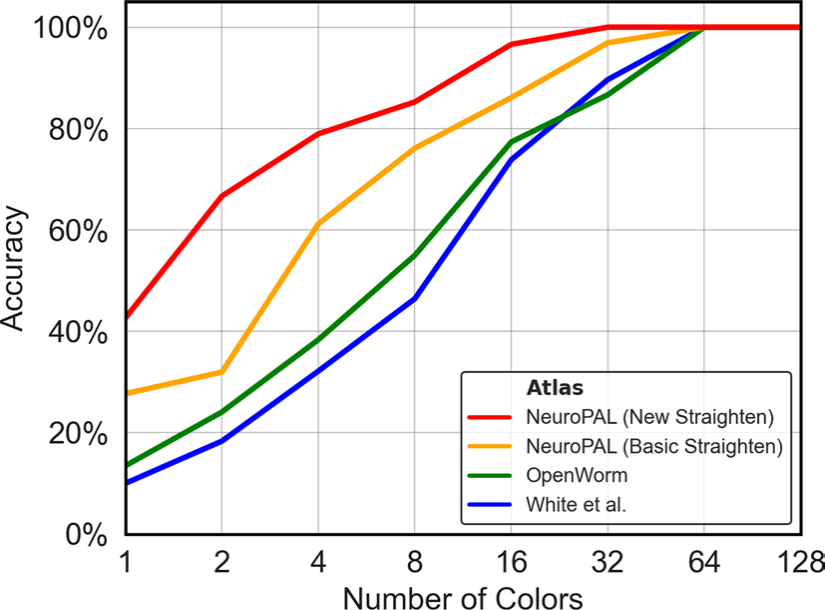
CPD Rigid Alignment: CPD Rigid was used to align each of the 7 manually labeled NeuroPAL individual worm point clouds to the tested atlases, after cropping of the heads and tails. The figure shows how the median accuracy for these 7 worms depends on the number of unique colors used. For each number of colors $n>1$, the colors were randomized and assigned to each neuron in both of the tested point clouds according to neuron identity, and CPD Rigid was run for each color such that it was only able to take into account the points that corresponded to the currently analyzed color

**Fig. 11:**
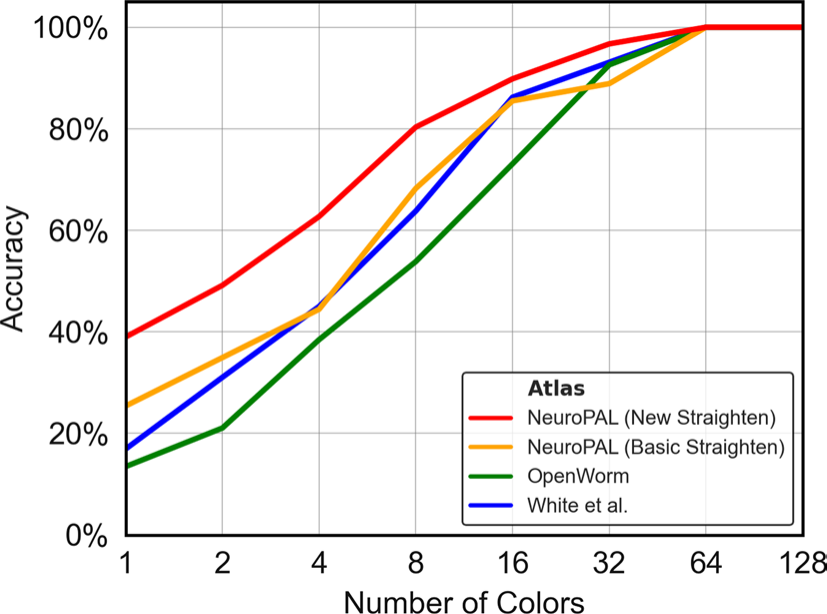
CGM Realistic Alignment: Same as previous figure, but for CPD Deformable alignment

**Fig. 12:**
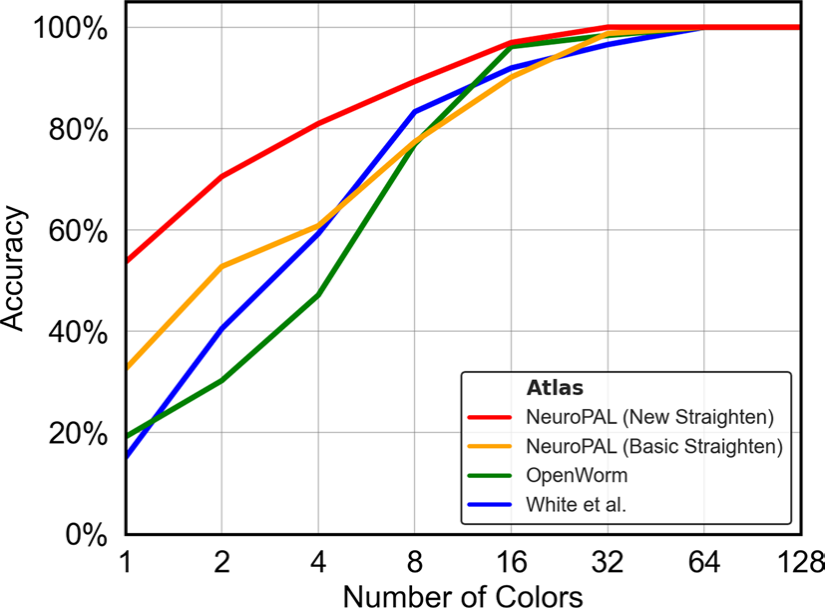
GM Realistic Alignment: Same as previous figure, but for GM Realistic alignment. Here the accuracy for the 7 worms is computed by averaging 40 runs each, and then the median is taken as in the two previous figures. For each number of colors $n>1$, the colors were randomized and assigned to each neuron in both of the tested point clouds according to neuron identity, and so GM Realistic now makes use of the *c* terms in Eq. (4)

We now test our NeuroPAL-based identification pipeline against previous atlas-based methods, in order to quantify their comparative performance, and also address the question of how many identification-assisting colors are necessary to achieve acceptable accuracy. Consider, for example, the case of aligning two point clouds, each consisting of the same 302 neurons. If every neuron in one point cloud was assigned the same unique distinguishable color as its corresponding neuron in the other (so that there are 302 distinct colors in all), any reasonable alignment algorithm exploiting color information should be able to produce 100% accurate alignment, since there is only one possible assignment for each neuron (its color twin). However, if we assigned 151 colors, each would correspond to two possible neuron identities. If we only assigned one single color to all neurons in each point cloud, an alignment algorithm would have 302 possible assignments for each neuron, and would have to rely on positional information alone. In Figs. 10, 11 and 12, we plot the performance of the three tested algorithms on our candidate atlases across a range of numbers of colors.

In addition, we include a version of the NeuroPAL atlas that has been straightened in a basic way as described by [24]. In these tests, all atlases were isotropically rescaled so as to occupy $x\in [0\,\upmu{\text{m}}, 800\,\upmu{\text{m}}]$, and to isolate the midbody the atlases were cropped at $x\in [200\,\upmu{\text{m}}, 700\,\upmu{\text{m}}]$. Then, alignments were performed using the same parameters as identified in Alignment performance on simulated data.

From the results, we have the following observations. Firstly, the NeuroPAL atlas outperforms the OpenWorm and White et al. atlases by a large margin, for all alignment methods, on mid-body data. This suggests that our NeuroPAL atlas combined with the circle straightening method enables more accurate identification. Secondly, comparing Figs. 12 and 10, we see that the GM Realistic method outperforms CPD methods across all colors and atlases, again demonstrating that our alignment method allows more accurate neuron identification. Thirdly, as the number of colors increases, the performance of all atlases increases, emphasizing the important role that NeuroPAL can play in enabling more accurate automated neuron identification for atlas creation.

## Conclusion

The main contributions of this paper are

1. a pipeline for constructing a 3D *C. elegans* atlas based on optically imaged neuron data,
2. an alignment method for identification of unlabeled *C. elegans* neurons using such an atlas, and
3. to the best of our knowledge, the most complete full-body *C. elegans* 3D positional neuron atlas, encapsulating positional variability derived from at least 7 animals per neuron.

We have presented tests suggesting that both our alignment algorithm and our pipeline-produced 3D atlas achieve higher identification accuracy than existing alternatives.

Many groups around the world are in the process of producing better imaging datasets so as to enable more promising investigation of myriad aspects of *C. elegans*, from neuronal activity to gene expression and cell-fate. We hope that, by delivering higher cell-type identification confidence, our atlas and others created using this method will help maximize the scientific value enabled by such functional imaging work.

## Supplementary Information

**Additional file 1.** This supporting information includes the optimal hyperparameters found for the alignment algorithms, and a list of all neuron positions in our newly assembled whole-worm atlas.

## Data Availability

All code and supporting data is available at (https://github.com/bluevex/elegans-atlas) upon publication.

## Abbreviations

*C. elegans*: Caenorhabditis elegans
EM: Electron micrograph
L4: Fourth larval stage
nm: Nanometer
2D: Two-dimensional
3D: Three-dimensional
GFP: Green fluorescent protein
CFP: Cyan fluorescent protein
YFP: Yellow fluorescent protein
GMM: Gaussian mixture model
CPD: Coherent point drift
GM: Generalized-mean
ID: Identification
Eq: Equation
