# Supplementary material for: Toward a more accurate 3D atlas of C. elegans neurons
Source: BMC Bioinformatics. 2022 May 28;23:195. doi: 10.1186/s12859-022-04738-3 (PMC9145532; doi:10.1186/s12859-022-04738-3)
Supplement: Supplementary file 1 — Additional file 1. This supporting information includes the optimal hyperparameters found for the alignment algorithms, and a list of all neuron positions in our newly assembled whole-worm atlas. [file 12859_2022_4738_MOESM1_ESM.pdf]

Supporting information

S1 Appendix. **Hyperparameter settings.** Here, we provide the hyperparameters chosen for the GM Realistic and CPD Deformable algorithms, which have been optimized on the experiments in Robustness to Cropping and applied to all experiments. For GM Realistic, the exponent γ in Eq. (4) is set to -5. We train for $T = 5000$ epochs with learning rate $\mathrm{lr} = 6.31 \times 10^{-4}$ which is reduced by a factor of 0.3 if the best validation loss does not improve for 100 consecutive epochs (30 epochs for the experiment in Robustness to Cropping). To prevent numerical errors from division by near-zero during optimization when $\left|\mathbf{r}_i^{(1)} - \mathbf{r}_j^{(2)}\right|$ is small (so that $\left|\mathbf{r}_i^{(1)} - \mathbf{r}_j^{(2)}\right|^\gamma$ is large, since $\gamma < 0$), we clamp its value by replacing $\left|\mathbf{r}_i^{(1)} - \mathbf{r}_j^{(2)}\right|^\gamma$ with $\min\left(\left|\mathbf{r}_i^{(1)} - \mathbf{r}_j^{(2)} + \epsilon\right|^\gamma, 10^{20}\right)$ with $\epsilon = 10^{-10}$. Moreover, we add a small regularization term that encourages the unlabeled point cloud to keep its original shape, by regularizing Eq. (4) as follows:

$$\ell_{GM}(\{\mathbf{r}_i^{(1)}\}, \{\mathbf{r}_j^{(2)}\}, \{c_i^{(1)}\}, \{c_j^{(2)}\}) = \sum_{j=1}^{n_2} \left(\frac{\sum_{i=1}^{n_1} \delta_{c_i^{(1)} c_j^{(2)}} \min\left(\left|\mathbf{r}_i^{(1)} - \mathbf{r}_j^{(2)} + \epsilon\right|^\gamma, 10^{20}\right)}{\sum_{i=1}^{n_1} \delta_{c_i^{(1)} c_j^{(2)}}} \right)^{\frac{1}{\gamma}} + \lambda \left|\left|\mathbf{A}^{(1)} - \mathbf{A}_0^{(1)}\right|\right|_F^2 \quad (7)$$

Here $\mathbf{A}^{(1)}$ is the matrix containing pairwise distances between each neuron pair in $\mathbf{r}^{(1)}$, and $\mathbf{A}_0^{(1)}$ is $\mathbf{A}^{(1)}$ at the start of training. $||\cdot||_F$ denotes Frobenius norm. For λ, we use an annealing procedure of $\lambda(\tau) = \lambda_0 + (\lambda_m - \lambda_0) \cdot \max(1, \frac{\tau}{T \cdot \eta})$ where τ is the epoch number, and λ_0, λ_m, η are hyperparameters. In the experiment at Testing alignment methods - Real Data, we use $N = 200$ deformation centers, with $\sigma = 0.2$ and initial amplitude $|\mathbf{d}_0|$ of $\mathbf{d}$ being 0.015. In experiment at Robustness to Cropping, we use $N = 50$ deformation centers, with $\sigma = 50$ and initial amplitude $|\mathbf{d}_0|$ of 0.1, due to different scenario and units. The detailed hyperparameters that we tuned, including search range and the chosen value, is provided in Table 1.

Table 1 below provides the hyperparameter setting. The parameters α and β are defined in [25]. For other hyperparameters, we use the default setting as in its implementation at https://github.com/siavashk/pycpd (as of Aug 1, 2020).

Hyperparameter	Value	Search Range
γ	-5	{-1,-2,-3,-4,-5,-6,-7,-8}
lr	3.19×10^{-3}	$[1 \times 10^{-6}, 0.5]$
T	5000	{1000,5000}
λ_0	0.443019	[0, 1]
λ_m	0.241575	[0, 1]
η	0.766147	[0, 1]
α	0.0771160	[0, 10]
β	191.03671	[0, 200]

Table 1: Hyperparameters for the GM Realistic method (first group) and CPD Deformable method (α, β).

S2 Appendix. **The NeuroPAL atlas, tabulated.** Here, we provide the neuron coordinates for the NeuroPAL atlas that we generated over the course of this work. The coordinates of these 300 neurons can also be downloaded at `https://github.`

com/bluevex/elegans-atlas. As explained in the text, the two CAN cells are not included in our atlas.

Table 2: Positions of aggregated NeuroPAL atlas, in microns.

Neuron	x	y	z
ADAL	94.34	0.03	10.31
ADAR	91.22	-1.43	-11.64
ADEL	87.97	-0.12	10.50
ADER	83.23	-0.58	-11.47
ADFL	54.18	8.48	7.00
ADFR	54.63	7.15	-8.88
ADLL	59.13	12.05	3.86
ADLR	58.80	11.38	-6.34
AFDL	53.37	5.99	7.49
AFDR	53.11	5.71	-9.19
AIAL	66.74	-4.67	0.66
AIAR	67.33	-4.73	-0.99
AIBL	55.49	2.53	5.95
AIBR	55.94	2.61	-6.63
AIML	79.31	-6.77	2.87
AIMR	70.59	-5.01	-3.48
AINL	68.22	6.62	9.69
AINR	67.38	7.01	-10.84
AIYL	71.42	-4.40	5.34
AIYR	59.43	-1.49	-1.12
AIZL	68.92	2.53	7.98
AIZR	67.51	1.72	-8.68
ALA	51.26	15.36	-2.16
ALML	337.49	11.79	-11.86
ALMR	313.28	19.99	10.89
ALNL	784.36	12.09	3.51
ALNR	786.60	12.15	-4.00
AQR	87.59	-3.56	-10.65
AS1	125.47	-9.54	1.86
AS10	656.89	-9.97	-1.18
AS11	721.64	-5.19	0.47
AS2	178.62	-14.41	2.54
AS3	241.85	-13.12	1.37
AS4	314.15	-15.93	0.51
AS5	373.84	-16.51	0.11
AS6	416.50	-13.72	0.36
AS7	480.05	-12.86	1.70
AS8	535.33	-13.19	-0.11
AS9	598.95	-11.42	-0.09
ASEL	61.30	5.43	8.57
ASER	60.94	5.42	-10.37
ASGL	59.98	8.66	7.01
ASGR	60.15	8.00	-8.22
ASHL	58.42	4.21	8.35
ASHR	57.83	3.81	-10.20
ASIL	63.34	10.14	5.80
ASIR	63.42	9.72	-7.16
ASJL	64.06	-0.02	5.64
ASJR	64.01	-1.08	-5.99
ASKL	54.73	12.00	4.44
ASKR	54.43	11.18	-6.32
AUAL	60.91	0.84	8.36
AUAR	60.76	0.22	-9.63
AVAL	50.20	6.70	8.41
AVAR	50.99	4.79	-9.13
AVBL	63.74	5.10	7.12
AVBR	62.69	4.83	-9.41
AVDL	65.34	5.35	9.11
AVDR	65.45	5.14	-10.59
AVEL	51.08	4.58	5.98
AVER	52.03	3.63	-6.09
AVFL	94.37	-7.80	3.49
AVFR	95.54	-8.59	0.49
AVG	121.12	-9.44	-1.76

Table 2 – continued

Neuron	x	y	z
AVHL	62.68	9.65	7.85
AVHR	62.82	8.96	-9.77
AVJL	65.08	7.13	8.47
AVJR	65.13	7.55	-9.59
AVKL	87.39	-5.94	3.82
AVKR	75.68	-5.64	-3.17
AVL	70.82	-4.87	0.37
AVM	286.36	-1.16	10.16
AWAL	57.58	6.57	5.85
AWAR	57.24	6.53	-7.97
AWBL	57.36	8.30	8.52
AWBR	57.40	8.10	-10.25
AWCL	55.82	3.00	9.03
AWCR	56.43	1.74	-9.51
BAGL	38.69	5.69	8.54
BAGR	39.43	4.06	-8.24
BDUL	186.23	7.64	-10.13
BDUR	185.33	11.51	7.87
CEPDL	49.58	13.62	3.70
CEPDR	50.28	12.90	-7.07
CEPVL	39.34	1.03	4.58
CEPVR	40.68	1.01	-4.42
DA1	129.53	-9.48	-1.52
DA2	190.41	-14.38	-0.02
DA3	266.33	-14.92	-0.37
DA4	347.38	-17.97	-0.59
DA5	460.64	-14.18	1.56
DA6	546.48	-13.87	-0.03
DA7	656.94	-9.31	-0.37
DA8	745.92	-0.22	-1.35
DA9	743.47	-1.67	-2.51
DB1	121.18	-10.32	-1.02
DB2	99.22	-9.21	0.23
DB3	178.69	-14.50	-0.77
DB4	304.79	-16.26	-0.36
DB5	413.08	-14.18	0.87
DB6	532.94	-14.57	-0.14
DB7	650.43	-9.69	-0.78
DD1	124.58	-9.32	0.77
DD2	224.86	-13.53	1.26
DD3	351.57	-15.60	1.38
DD4	476.91	-14.26	2.44
DD5	591.74	-11.87	-0.19
DD6	736.31	-1.72	-0.59
DVA	770.84	10.96	-1.61
DVB	769.29	11.37	0.98
DVC	772.86	11.85	1.55
FLPL	81.11	0.02	10.58
FLPR	74.92	0.23	-11.36
HSNL	462.53	-1.78	-9.81
HSNR	452.01	-3.33	10.54
I1L	0.71	7.43	4.12
I1R	0.00	6.00	-2.71
I2L	13.08	4.85	7.23
I2R	13.82	3.39	-6.00
I3	18.92	13.57	-0.96
I4	71.62	10.84	-0.58
I5	72.18	-2.79	-2.56
I6	75.59	10.21	4.19
IL1DL	32.71	13.12	2.18
IL1DR	33.88	12.22	-4.59
IL1L	30.48	7.23	6.98
IL1R	30.97	5.64	-6.96
IL1VL	28.94	1.64	3.72
IL1VR	29.64	0.96	-1.72
IL2DL	24.21	12.34	4.34
IL2DR	26.43	12.45	-6.68
IL2L	29.90	7.93	9.15
IL2R	30.16	6.61	-8.80
IL2VL	25.35	2.14	5.06

Table 2 – continued

Neuron	x	y	z
IL2VR	28.36	0.63	-4.16
LUAL	774.32	6.28	5.16
LUAR	773.38	6.23	-5.35
M1	75.48	13.67	-6.40
M2L	69.41	2.97	3.49
M2R	69.57	4.17	-5.89
M3L	22.36	5.63	4.81
M3R	22.75	5.14	-2.91
M4	23.55	12.29	-0.69
M5	86.79	9.68	7.12
MCL	14.37	5.92	4.27
MCR	14.19	5.25	-3.51
MI	16.72	14.61	-1.03
NSML	15.78	4.53	7.54
NSMR	16.66	4.10	-5.46
OLLL	31.34	10.05	6.06
OLLR	32.56	8.59	-7.21
OLQDL	37.55	14.22	2.16
OLQDR	38.70	13.39	-5.63
OLQVL	36.66	3.93	6.16
OLQVR	37.26	3.11	-5.90
PDA	754.21	0.20	-2.03
PDB	748.26	-0.57	-3.40
PDEL	561.79	12.14	-7.53
PDER	555.40	11.49	10.46
PHAL	770.37	3.83	5.96
PHAR	770.06	4.14	-5.70
PHBL	773.56	5.55	6.00
PHBR	773.40	5.46	-6.33
PHCL	782.89	5.39	4.74
PHCR	782.27	5.49	-4.85
PLML	794.74	5.35	3.99
PLMR	795.65	5.12	-4.57
PLNL	770.37	3.83	5.29
PLNR	773.01	4.33	-4.55
PQR	776.96	4.86	5.20
PVCL	777.42	6.51	4.85
PVCR	776.14	5.98	-5.19
PVDL	555.97	12.66	-8.11
PVDR	540.27	11.60	7.00
PVM	556.10	9.96	-7.73
PVNL	794.35	6.90	3.24
PVNR	800.00	9.67	-2.87
PVPL	731.85	-3.27	1.07
PVPR	737.11	-1.81	-2.55
PVQL	766.78	6.02	6.41
PVQR	767.28	6.11	-5.59
PVR	786.71	8.07	-3.88
PVT	731.25	-2.90	-1.85
PVWL	782.97	7.52	4.18
PVWR	784.17	7.74	-4.13
RIAL	51.21	9.98	5.75
RIAR	51.66	9.13	-7.32
RIBL	62.04	3.07	6.02
RIBR	61.68	2.30	-6.76
RICL	70.85	0.28	8.03
RICR	69.97	-0.20	-8.23
RID	48.01	15.71	-2.99
RIFL	99.02	-8.14	2.31
RIFR	106.41	-9.05	-1.71
RIGL	114.67	-8.73	1.71
RIGR	117.71	-8.93	0.23
RIH	47.05	-0.69	-0.08
RIML	63.59	-0.63	4.45
RIMR	62.64	-1.99	-4.65
RIPL	35.30	7.97	6.37
RIPR	35.72	7.11	-6.56
RIR	54.10	0.76	1.40
RIS	87.93	-7.68	0.35
RIVL	64.03	11.92	4.43

Table 2 – continued

Neuron	x	y	z
RIVR	64.38	11.31	-7.21
RMDDL	55.78	0.20	3.91
RMDDR	56.06	-0.46	-2.32
RMDL	50.76	3.32	8.18
RMDR	52.31	1.87	-7.88
RMDVL	47.33	7.10	9.76
RMDVR	48.66	5.29	-10.21
RMED	40.14	15.50	-2.72
RMEL	38.03	8.71	7.36
RMER	38.76	7.16	-7.87
RMEV	44.66	-0.27	1.17
RMFL	58.12	-3.06	1.77
RMFR	57.51	-2.40	0.07
RMGL	98.07	-0.45	9.18
RMGR	96.44	-1.47	-10.33
RMHL	58.16	-0.78	3.15
RMHR	56.46	-1.22	-0.14
SAADL	53.44	-0.90	1.45
SAADR	50.27	-0.86	-0.39
SAAVL	49.05	9.73	6.88
SAAVR	49.34	8.24	-8.03
SABD	121.86	-9.41	-1.80
SABVL	86.35	-7.78	2.35
SABVR	80.61	-7.60	-2.17
SDQL	559.25	8.99	-7.45
SDQR	241.27	20.00	9.89
SIADL	55.68	-1.65	2.21
SIADR	53.27	-2.23	-1.59
SIAVL	66.79	-3.37	3.33
SIAVR	69.64	-4.92	-2.10
SIBDL	53.70	1.28	6.25
SIBDR	54.50	1.43	-6.72
SIBVL	59.54	-2.49	2.74
SIBVR	53.46	-1.56	-1.27
SMBDL	59.79	-1.85	2.44
SMBDR	60.22	-1.45	-1.90
SMBVL	62.73	-2.88	3.55
SMBVR	67.29	-4.46	-1.61
SMDDL	51.10	-0.48	2.46
SMDDR	54.10	0.01	-2.69
SMDVL	51.19	10.92	7.29
SMDVR	51.03	9.15	-9.28
URADL	30.69	12.53	4.50
URADR	31.32	11.24	-6.46
URAVL	33.57	2.35	4.74
URAVR	34.97	2.42	-3.85
URBL	34.05	7.53	8.41
URBR	34.27	6.21	-8.65
URXL	50.65	13.05	2.27
URXR	50.87	12.30	-5.18
URYDL	34.36	12.32	4.64
URYDR	35.22	10.80	-6.94
URYVL	32.70	5.12	6.71
URYVR	33.10	3.42	-6.05
VA1	108.33	-9.03	1.87
VA10	633.31	-10.82	-0.85
VA11	690.16	-7.21	0.31
VA12	737.02	-2.29	1.89
VA2	155.90	-12.66	0.30
VA3	209.48	-14.35	-0.15
VA4	274.69	-16.08	0.65
VA5	337.32	-16.96	1.41
VA6	407.98	-13.73	2.07
VA7	459.17	-14.93	2.93
VA8	505.74	-14.30	0.25
VA9	572.47	-13.18	-0.53
VB1	105.50	-8.78	1.39
VB10	581.55	-13.68	-0.70
VB11	640.50	-9.69	-0.66
VB2	85.76	-8.14	1.61

Table 2 – continued

Neuron	x	y	z
VB3	162.52	-13.05	0.50
VB4	217.31	-14.80	-0.10
VB5	287.47	-16.37	0.62
VB6	342.69	-16.78	1.46
VB7	405.49	-14.51	1.10
VB8	467.40	-15.05	2.99
VB9	524.97	-14.45	-0.04
VC1	224.54	-13.22	-0.14
VC2	294.26	-17.48	0.50
VC3	383.44	-16.60	1.40
VC4	441.76	-11.49	0.18
VC5	455.55	-8.49	0.58
VC6	530.44	-12.14	0.58
VD1	113.77	-8.15	1.06
VD10	602.94	-11.22	-0.04
VD11	660.86	-8.57	-0.53
VD12	727.56	-3.93	0.66
VD13	744.41	-0.35	-3.30
VD2	132.47	-9.95	-1.74
VD3	206.99	-13.59	-0.45
VD4	263.69	-14.60	1.18
VD5	319.93	-16.50	0.56
VD6	383.75	-15.85	0.96
VD7	431.61	-14.25	-1.05
VD8	482.54	-13.22	2.60
VD9	548.63	-13.48	-0.01
